# Better safe than sorry: the unexpected drought tolerance of a wetland plant (*Cyperus alternifolius* L.)

**DOI:** 10.1111/ppl.70027

**Published:** 2024-12-26

**Authors:** Lucia Nadia Biruk, Martina Tomasella, Francesco Petruzzellis, Andrea Nardini

**Affiliations:** ^1^ Dipartimento di Scienze della Vita Università di Trieste Trieste Italia; ^2^ Dipartimento di Biologia Università di Padova Padova Italia

## Abstract

A common assumption of plant hydraulic physiology is that high hydraulic efficiency must come at the cost of hydraulic safety, generating a trade‐off that raises doubts about the possibility of selecting both productive and drought‐tolerant herbaceous crops. Wetland plants typically display high productivity, which requires high hydraulic efficiency to sustain transpiration rates coupled to CO_2_ uptake. Previous studies have suggested high vulnerability to xylem embolism of different wetland plants, in line with expected trade‐offs. However, some hygrophytes like *Cyperus alternifolius* L. can also experience prolonged periods of low water levels leading to substantial drought stress. We conducted an in‐depth investigation of this species' hydraulic safety and efficiency by combining gas exchange measurements, hydraulic measurements of leaf hydraulic efficiency and safety, optical measurements of xylem vulnerability to embolism, and determination of cell turgor changes under drought. Our data confirm the high hydraulic efficiency of this wetland species, but at the same time, reveal its surprising drought tolerance in terms of turgor loss point and critical water potential values inducing xylem embolism and hydraulic failure, which were well below values inducing turgor loss and full stomatal closure. *C. alternifolius* emerges as a highly productive plant that is also well‐equipped to tolerate drought via a combination of early stomatal closure and delayed onset of hydraulic damage. The species might represent a model plant to develop crops combining two of the most desirable traits in cultivated plants, i.e., high yield and significant drought tolerance.

## 1 INTRODUCTION

Water deficit is one of the most significant abiotic constraints to the growth, productivity, and survival of wild and cultivated plants (Dietz et al., 2021; Liu et al., 2021), and both drought frequency and severity are predicted to increase because of climate change (Chiang et al., 2021). A large body of scientific literature has investigated the impacts of extreme and/or recurrent drought on plant performance to understand and predict negative effects on natural ecosystems and to develop strategies for breeding crop varieties displaying high productivity and drought tolerance.

Plant responses to water shortage depend on species‐specific functional traits, with water potential at turgor loss point and vulnerability to xylem embolism largely dictating stomatal response and probability of survival under drought progression (Nardini et al., 2013; Alvarez‐Cansino et al., 2021; Petruzzellis et al., 2022). The turgor loss point (Ψ_tlp_) corresponds to the leaf water potential (Ψ_leaf_) at which cells lose turgor and undergo plasmolysis, with possible damage to membranes and increasing risks for cell survival (Trueba et al., 2019; Trifilò et al., 2023). Lower Ψ_tlp_ values allow plants to maintain stomata open for longer periods under water shortage, thus extending the time available for net carbon gain while delaying the onset of plasmolysis (Blackman, 2018). There is a large inter‐specific variability of Ψ_tlp_ at different ecological scales (Nardini et al., 2012; Bartlett et al., 2012), with species thriving in water‐limited habitats typically displaying more negative values compared to those occupying mesic to humid environments (Tordoni et al., 2022). Complete stomatal closure generally occurs at leaf water potential values close to Ψ_tlp_, but under prolonged drought, the residual water loss at cuticular or bark level (Wolfe, 2020; Wang et al., 2024) can force water potential to reach critical values triggering air seeding in xylem conduits, leading to xylem embolism and potential failure of the long‐distance water transport in the plant (Nardini et al., 2013). Vulnerability to xylem embolism is generally quantified based on vulnerability curves (VCs), i.e., plots of stem, leaf, or root xylem hydraulic efficiency (or accumulation of embolism events) as a function of the progressive drop of water potential under natural or experimental drought conditions (Cochard et al., 2013). VCs can be used to extrapolate synthetic indices of vulnerability to xylem embolism, like the water potential inducing a 50% loss of xylem hydraulic efficiency (Ψ_50_). Just like tolerance to turgor loss described by Ψ_tlp_, also the vulnerability to xylem embolism displays large inter‐specific and ecological variability, so that species occupying xeric habitats are typically characterized by more negative Ψ_50_ compared to those dominating mesic and humid habitats (Pockman & Sperry, 2000; Trueba et al., 2017).

Several studies have revealed that Ψ_tlp_ and Ψ_50_ are generally correlated across species (e.g. Nardini & Luglio, 2014; Chen et al., 2021). This likely reflects the need to coordinate symplastic and apoplastic drought tolerance, but at the leaf level this correlation can also derive on mechanistic bases. This is especially the case when considering the reduction of hydraulic efficiency induced by drought stress at the extra‐vascular level (Trifilò et al., 2016; Trifilò et al., 2021; Nardini, 2022) that can be induced by turgor loss and cell shrinking (Scoffoni et al., 2014). The clear adaptive role of Ψ_tlp_ and Ψ_50_ in species‐specific drought tolerance, as revealed by physiological considerations and ecological evidence, makes these traits an attractive target for breeding crops with enhanced capacity to survive water shortage (Mart et al., 2016; Lamarque et al., 2023). Unfortunately, both these traits might potentially trade‐off with species‐specific productivity and growth rates, so that species with lower Ψ_tlp_ and Ψ_50_ also display lower biomass production and growth rates and vice versa (Max et al., 2023), although contrasting results emerged in the literature in this respect (e.g., Gleason et al., 2016; Xiong & Flexas, 2022). The trade‐off between hydraulic safety and productivity might depend on the substantial amounts of energy and carbon invested by plants to achieve high resistance against the risks of turgor loss and hydraulic failure. This trend might arise from several metabolic or structural requirements associated with both functional traits.

Lowering Ψ_tlp_ requires the accumulation of solutes in cells (Bartlett et al., 2012), implying energy and carbon costs for synthesizing sugars and other compatible osmolytes or fueling membrane transport processes leading to the accumulation of inorganic ions. Low Ψ_tlp_ values are also often associated with small cells mechanically reinforced by thick cell walls (Ding et al., 2014). This feature likely limits cell shrinkage under plasmolysis and possibly prevents cell damage upon full hydration, because the low osmotic potential required to adjust Ψ_tlp_ unavoidably leads to high turgor pressures under well‐watered conditions (Nardini, 2022). Besides the substantial carbon costs associated with such anatomical modifications, thick cell walls also limit CO_2_ diffusion from the atmosphere to the chloroplasts, thus lowering maximum photosynthetic capacity and potential productivity (Evans, 2021).

The achievement of low Ψ_50_ values also implies carbon costs and typically leads to reduced photosynthetic performance. Xylem highly resistant to embolism formation is characterized by relatively small conduits (Isasa et al., 2023) with thickened cell walls associated with increased thickness of pit membranes (Li et al., 2016) and a possible reduction of the risk of implosion and collapse under sustained tension (Hacke et al., 2001). Narrow xylem conduits are hydraulically much less efficient than larger ones (Hacke et al., 2017), thus requiring a higher number of conduits per unit cross‐sectional area to partly compensate for the reduction of water flow rates (Pfautsch et al., 2016). Further hydraulic limitations derive from thick inter‐vessel pit membranes designed to limit the spread of the gas phase in the xylem network (Levionnois et al., 2022). These anatomical and ultrastructural modifications lead to high carbon costs and reduced water supply to the foliage, finally limiting gas exchange rates, photosynthetic capacity, and growth rates (Nardini & Salleo, 2000; Nardini, 2002; Santiago et al., 2004).

Most of the currently available evidence for the role of Ψ_tlp_ and Ψ_50_ in drought resistance and related trade‐offs with photosynthetic productivity are based on studies focused on woody plants, while herbaceous ones have been subjected to much less scrutiny in this respect (Casolo et al., 2015; Lens et al., 2016; Nolf et al., 2016; Dória et al., 2019), with the notable exception of some economically important crops (Li et al., 2009; Gleason et al., 2017; Savi et al., 2017; Ahmad et al., 2018; D'Incà et al., 2024). Even less is known about herbaceous wetland plants, which are expected to display high gas exchange rates and photosynthetic productivity to successfully compete in these high‐resource environments (Junk & Piedade, 1993; Saunders et al., 2014). According to the trade‐offs described above, this should lead to relatively high vulnerability to drought stress (i.e., high values of Ψ_tlp_ and Ψ_50_), as indeed suggested by the very few studies that investigated these traits in this functional group (Zhou et al., 2013; Alemán‐Sancheschúlz et al., 2020). On the other hand, several wetland plants inhabiting semi‐arid zones experience frequent water availability fluctuations as a function of seasonal changes in water levels in rivers and lakes (Miller & Zedler, 2003; Vivian et al., 2014). This might suggest that some of them have also developed drought tolerance traits leading to an unusual and unexpected combination of high hydraulic efficiency and high hydraulic safety (Yao et al., 2021). *Cyperus alternifolius* L. is a wetland plant that is particularly interesting for different reasons. Native to Africa, Madagascar, and the Arabian Peninsula, it grows primarily in tropical regions where it can experience frequent water availability fluctuations, thus withstanding even relatively intense drought spells. The species is also widely naturalized in several different areas of the globe, where it has become invasive, even colonizing dried‐out riverbeds and ravines (Veerloove, 2014). The species is also known for its high primary productivity and efficiency in removing pollutants, which makes it a good candidate for urban nature‐based solutions for hydrologic regulation and phytoremediation (Tuttolomondo et al., 2015).

In this study, we report a detailed analysis of the hydraulic efficiency and safety of *C. alternifolius* as a potential model plant combining high potential productivity with substantial drought tolerance, and we compare our findings with recent reports suggesting extremely high drought vulnerability of other wetland plants.

## 2 MATERIALS AND METHODS

### 2.1 Plant material

All measurements were performed between June and September 2023 on 4–6 months old plants of *Cyperus alternifolius* L. Individuals were propagated from rhizomes (3–5 cm long segments) collected from a single large plant growing in the greenhouse of the Botanical garden of the University of Trieste, Italy (45.661 N, 13.795 E). Rhizomes were placed in 1 L pots filled with light‐expanded clay aggregate and grown outdoors while keeping the pots submerged in water. Experiments involved a total of 15 different plants, each presenting several leaf‐bearing culms with a height of approximately 70 cm.

### 2.2 Leaf water potential isotherms

Leaf water potential isotherms, also known as pressure‐volume curves (Tyree & Hammel, 1972), were measured and elaborated to estimate leaf osmotic potential at full turgor (π_0_), water potential at turgor loss point (Ψ_tlp_), bulk modulus of elasticity (ε, calculated over the whole turgor range), apoplastic water fraction (AWF), and leaf capacitance (C_leaf_). This last variable was calculated based on the slope of the relationship between Ψ_leaf_ and the water loss before (C_leaf___FT_) and after Ψ_tlp_ (C_leaf___TLP_) and normalized by the leaf surface area (A_leaf_). Both values were used to calculate K_leaf_ according to equation 1 (see next section).

In the early morning, culms were cut under water and rehydrated in the laboratory for 1–2 h to full turgor and checked on the basis of sequential water potential measurements. Then, a leaf was cut for each culm (five replicates), immediately wrapped in plastic film and inserted into a Scholander pressure chamber (Mod. 1505D, PMS instrument co.). The initial Ψ_leaf_ was recorded and the leaves were immediately weighed using a precision balance. During progressive dehydration, leaf water loss and Ψ_leaf_ were measured at different time points. Measurements were continued until the relationship between −1/Ψ_leaf_ and water loss became linear (R^2^ > 0.95) for a minimum of four to five consecutive experimental points. At the end of each experiment, the leaf was scanned to measure A_leaf_ using ImageJ software (version 1.54f, NIH), and finally oven‐dried at 70° C for two days to measure leaf dry weight (DW). These two variables were used to estimate some of the variables mentioned above as well as the specific leaf area (SLA) as the ratio between A_leaf_ and DW.

### 2.3 Whole leaf hydraulic efficiency and vulnerability

Leaf hydraulic conductance (K_leaf_) at different dehydration levels was measured using the rehydration kinetic technique (Brodribb & Holbrook, 2003), i.e. by the change in Ψ_leaf_ before (Ψ_leaf_i_) and after (Ψ_leaf_f_) a rehydration period of known duration (*t*). Based on values of C_leaf_, derived from leaf water potential isotherms, it was possible to calculate K_leaf_ as:

(1) $$K_{\text{leaf}}=C_{\text{leaf}*}\frac{\ln\;\left(\Psi_{\text{leaf}\_i}/\Psi_{\text{leaf}\_f}\right)}{t}$$

C_leaf___FT_ or C_leaf___TLP_ were used in the equation when Ψ_leaf_i_ was above or below Ψ_tlp,_ respectively (Brodribb & Holbrook, 2003).

Culms were sampled in the morning and rehydrated to full turgor. Then they were bench‐dehydrated until Ψ_leaf_ reached values between −0.5 and − 2.5 MPa, i.e., until the rehydration capacity of leaves was severely impaired and K_leaf_ approached 0. The culms were placed in a plastic bag in the dark for 20–40 min to prevent water loss through transpiration and allow equilibration of the water potential across all leaves. Then two leaves were cut, immediately wrapped in plastic film and used to estimate Ψ_leaf_i_. If the difference in Ψ_leaf_ between the two leaves was greater than 0.1 MPa, the culm was discarded. A third leaf was cut with a razor blade while its base was immersed in distilled water and allowed to rehydrate for a time (t) of 10–40 s, depending on Ψ_leaf_i_ values. The leaf was placed in a plastic bag for 1 min before measuring Ψ_leaf_f_. In all cases, Ψ_leaf_ was measured with a Scholander pressure chamber. The maximum K_leaf_ was calculated as the average of values recorded for well‐hydrated leaves (Ψ_leaf_ > −0.7 MPa). A whole leaf hydraulic vulnerability curve was constructed by plotting the values of K_leaf_ obtained from leaves with different dehydration levels against the corresponding Ψ_leaf_i_. From this, Ψ_50_k_ was calculated as the Ψ_leaf_ at which the leaves lose 50% of the maximum K_leaf_.

### 2.4 Optical vulnerability curves of leaf vein xylem

The occurrence of leaf xylem embolism events under drought stress was monitored using the optical vulnerability (OV) method (Brodribb et al., 2016). Well‐hydrated plants were gently removed from pots and dehydrated under laboratory conditions. Before starting the dehydration, a fully expanded leaf was enclosed in a 3D‐printed clamp containing an 8‐megapixel camera and LED lights connected to a Raspberry Pi 4 computer. Images were captured using transmitted light every 5 min during plant dehydration until no further embolism events were observed for at least 3 h. Image analysis was carried out using ImageJ to quantify the embolized pixel area (see https://www.opensourceov.org/ for more information on the stages of image acquisition, processing and analysis). Simultaneously to image capturing during dehydration, we measured the water potential in neighbouring leaves belonging to the same culm. Initially, measurements were performed with a pressure chamber, but this instrument did not allow the measurement of Ψ_leaf_ < −4.5 MPa in the study species due to the crushing of tissues when very dehydrated leaves were sealed in the chamber. Hence, we decided to measure water potential using three different instruments in three sets of experiments:

1. Scholander pressure chamber (1505D, PMS instrument co.): five to ten leaves per plant were wrapped in plastic film and cut to measure Ψ_leaf_ at different time intervals.
2. Dewpoint hygrometer (WP4C, METER Group): five to ten leaves per plant were cut at different time intervals and perforated with metal needles. Then, three pieces about 2 cm long were re‐cut and inserted in the sample holder of the instrument. Ψ_leaf_ was measured by the continuous reading mode until the sample had reached vapor equilibrium (nearly 60 min).
3. Leaf psychrometer (PSY1‐LEAF, ICT International): a single leaf adjacent to the one used for the OV method was chosen, and the cuticle of a small portion was abraded with fine‐grit sandpaper. In this portion the psychrometer was attached to the leaf and the water potential was measured every 30 min.

The optical vulnerability curve was constructed by plotting the values of the percentage of xylem embolized area (i.e., cumulative embolized area / total embolized area) at different dehydration levels against the corresponding Ψ_leaf_. From this, Ψ_50_xe_ was calculated as the Ψ_leaf_ values inducing 50% of embolized xylem area.

In addition, the vein density (VD) and the embolized vein density (ED) were calculated as the length of the veins or total embolized veins per unit leaf area, respectively. In the first case, five fresh leaves were cleared, dehydrated and stained as described by Petruzzellis et al. (2021). Then, images of the leaves were captured with a digital camera connected to an optical microscope (total magnification 40X). In the second case, two images were considered: the first picture was captured using the OV method and a mask was created with all the embolized events using the ImageJ software with the Fiji package and the OSOV toolbox. The images were used to determine the length of embolized veins and the sample leaf area with ImageJ.

### 2.5 Measurements of gas exchange responses to drought

To determine the relationship between stomatal aperture and leaf water status, the leaf conductance to water vapour (g_leaf_) and Ψ_leaf_ were measured almost simultaneously using a porometer (Mod. LI‐600, LI‐COR Environmental) and a Scholander pressure chamber, respectively. Five plants were carefully removed from the pots to speed up dehydration. Three of them were allowed to dehydrate (drought treatment) under field conditions, while the remaining two plants were kept in water (control). During dehydration, air temperature oscillated between 25 and 30°C, while relative humidity ranged from 32 to 41%. Maximum photosynthetic photon flux density was 1140 μmol m^−2^ s^−1^. Fully expanded leaves were selected from each plant, and g_leaf_ was measured at different time intervals in different leaves, selecting the central portion of the leaf blade, on the abaxial surface. After each g_leaf_ measurement, the leaf was detached and immediately wrapped in plastic film to prevent water loss, and the corresponding Ψ_leaf_ was measured. Measurements were taken every hour between 9.00 and 14.00 h on two consecutive days until full stomatal closure, i.e. when g_leaf_ reached values lower than 5% of those measured in control plants.

A stomatal response curve was constructed by plotting the values of g_leaf_ obtained from leaves with different dehydration levels against the corresponding Ψ_leaf_ in order to calculate the water potential at which 95% reduction of leaf conductance to water vapour occurred (Ψ_g_95_). Additionally, the stomatal safety margin (SSM) was calculated to estimate the degree of conservatism in the plant hydraulic strategy, as the difference between Ψ_g_95_ and Ψ_50_xe_ (Skelton et al., 2015).

### 2.6 Data analysis

Whole leaf hydraulic vulnerability was quantified by calculating the Ψ_leaf_ inducing 50% loss of maximum hydraulic conductance (Ψ_50_k_), using the fitcond function in the “fitplc” R package (v 4.4.0, R Core Team 2024; Duursma & Choat, 2017). Specifically, the hydraulic vulnerability curve obtained as previously described was fitted using a sigmoidal model, and the 95% confidence intervals (CIs) were calculated through the bootstrap procedure (*n* = 1000). R^2^ was calculated using the rsquare function in the modelr R package (Wickham, 2023).

A similar analysis was performed to elaborate leaf OV vulnerability curves, in order to calculate Ψ_leaf_ corresponding to 50% of the embolized xylem area (Ψ_50_xe_). Specifically, one independent OV curve was calculated for each method used to measure Ψ_leaf_, i.e. pressure chamber, dewpoint hygrometer, and leaf psychrometer. Then, a Weibull model (Ogle et al., 2009) was calculated for each vulnerability curve using the fitplc function in the “fitplc” R package, and Ψ_50_xe_ values and associated 95% CIs were calculated as explained above. Additionally, an OV vulnerability curve was fitted pooling together the data obtained with the three methods used to measure Ψ_leaf_ to calculate the overall Ψ_50_xe_ value and associated 95% CIs. Differences in Ψ_50_xe_ values obtained with the three methods were considered statistically significant when CIs did not overlap. R^2^ values of each OV curve were calculated as indicated above.

A custom‐made bootstrap procedure, similar to the one proposed in Trifilò et al. (2023), was applied to calculate the Ψ_leaf_ inducing the loss of 95% of the maximum g_leaf_ value measured in control plants (Ψ_g_95_). First, data points were resampled at random with replacement, and a log‐logistic model was fitted using the drm function in the drc R package (Ritz et al., 2015). Then, Ψ_g_95_ was estimated using the approx function in the “stats” R package. This procedure was repeated 999 times, after which the average Ψ_g_95_ value and associated 95% CIs were calculated.

## 3 RESULTS

Based on leaf water potential isotherms, we calculated different water relations variables correlated to species‐specific drought tolerance (Table 1). Data analysis revealed that the turgor loss point (Ψ_tlp_) of *C. alternifolius* averaged at −1.8 MPa, while osmotic potential at full turgor (π_0_) and modulus of elasticity (ε, calculated on the basis of total RWC) averaged at −1.5 MPa and 17.5 MPa, respectively. When calculated based on symplastic RWC changes, ε turned out to be 10.5 ± 2 MPa. Saturated water content (SWC) was 2.4 g g^−1^, while bulk leaf capacitance was 0.37 and 1.49 mol m^−2^ MPa^−1^ in the turgid range (C_leaf_FT_) and after the turgor loss point (C_leaf_TLP_), respectively. The specific leaf area (SLA) averaged 18.9 mm^2^ mg^−1^. Total vein density was 6.2 mm mm^−2^, mostly contributed by parallel veins (5.6 mm mm^−2^), while cross veins had an average density of 0.6 mm mm^−2^.

**TABLE 1:** List of anatomical and physiological variables measured in leaves of *Cyperus alternifolius*, with relative abbreviations, units and mean values ± SD.

Variable	Mean ± SD
Saturated water content (SWC), g g^−1^	2.4 ± 0.2
Osmotic potential at full turgor (π_0_), MPa	−1.5 ± 0.1
Turgor loss point (Ψ_tlp_), MPa	−1.8 ± 0.1
Apoplastic water fraction (AWF), %	38.6 ± 8.0
Relative water content at Ψ_tlp_ (RWC_TLP_), %	89.7 ± 2.6
Modulus of elasticity (ε), MPa	17.5 ± 4.0
Leaf capacitance at full turgor (C_leaf_FT_), mol m^−2^ MPa^−1^	0.37 ± 0.09
Leaf capacitance at Ψ_tlp_ (C_leaf_TLP_), mol m^−2^ MPa^−1^	1.49 ± 0.19
Leaf surface area (A_leaf_), mm^2^	69.7 ± 21.1
Specific leaf area (SLA), mm^2^ mg^−1^	18.9 ± 1.09
Total vein density (VD_total_), mm mm^−2^	6.2 ± 0.3
Parallel vein density (VD_parallel_), mm mm^−2^	5.6 ± 0.3
Cross‐vein density (VD_cross_), mm mm^−2^	0.6 ± 0.1

**FIGURE 1:**
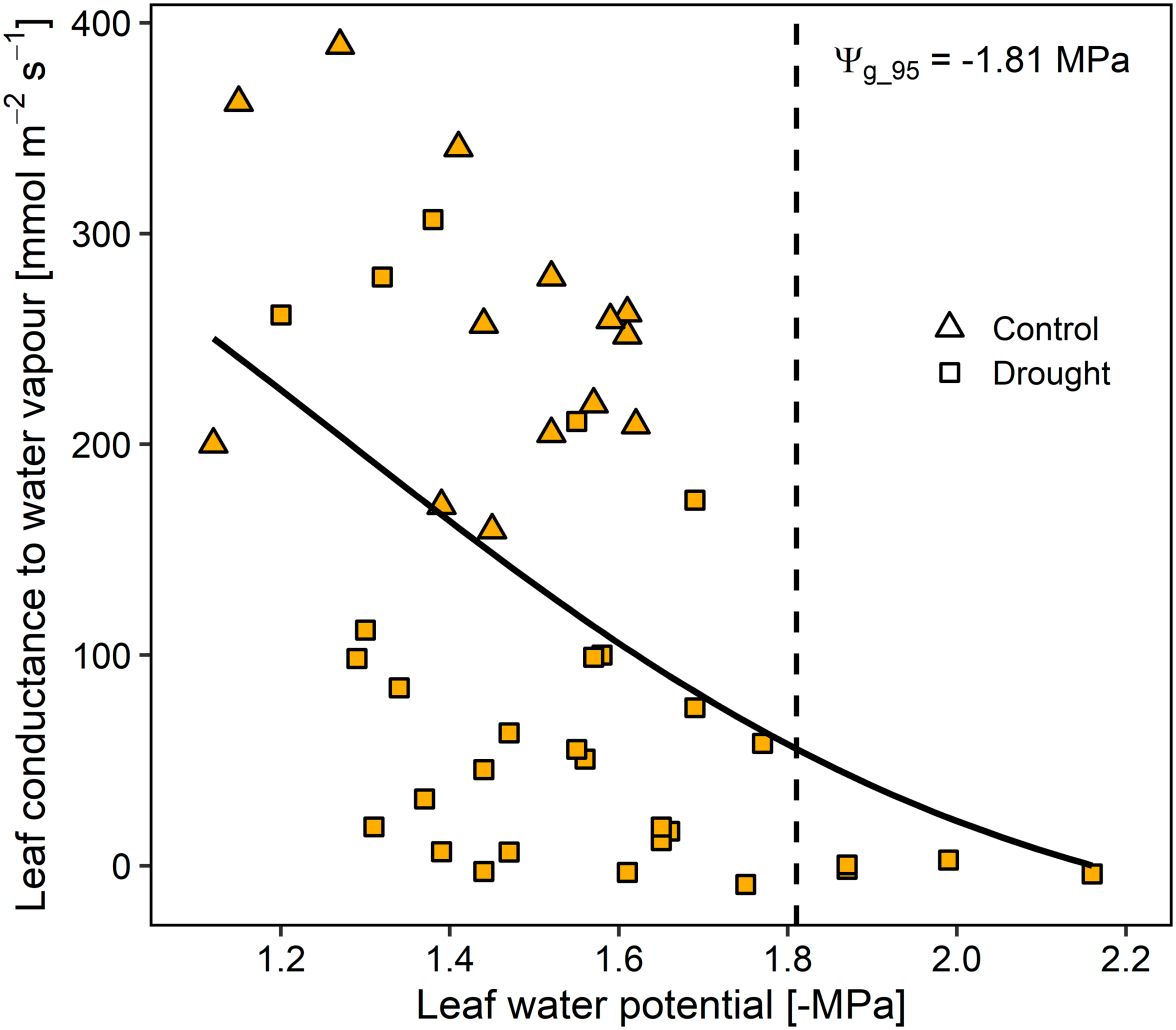
Changes in leaf conductance to water vapour as a function of leaf water potential in well‐watered plants (Control, triangles) as well as in plants deprived of water and subjected to progressive water stress (Drought, squares). The solid line represents the interpolation of experimental points, while the dashed vertical line indicates the leaf water potential value inducing 95% reduction of leaf conductance to water vapour compared to the maximum value (Ψ_g_95_).

Well irrigated plants displayed high values of leaf conductance to water vapour, up to 400 mmol m^−2^ s^−1^, at leaf water potential values of about −1.0 MPa. During dehydration, stomata progressively closed until a minimum g_leaf_ of 5–10 mmol m^−2^ s^−1^ at Ψ_leaf_ < −1.8 MPa (Figure 1). Ψ_g_95_ was reached when Ψ_leaf_ averaged at −1.81 MPa.

**FIGURE 2:**
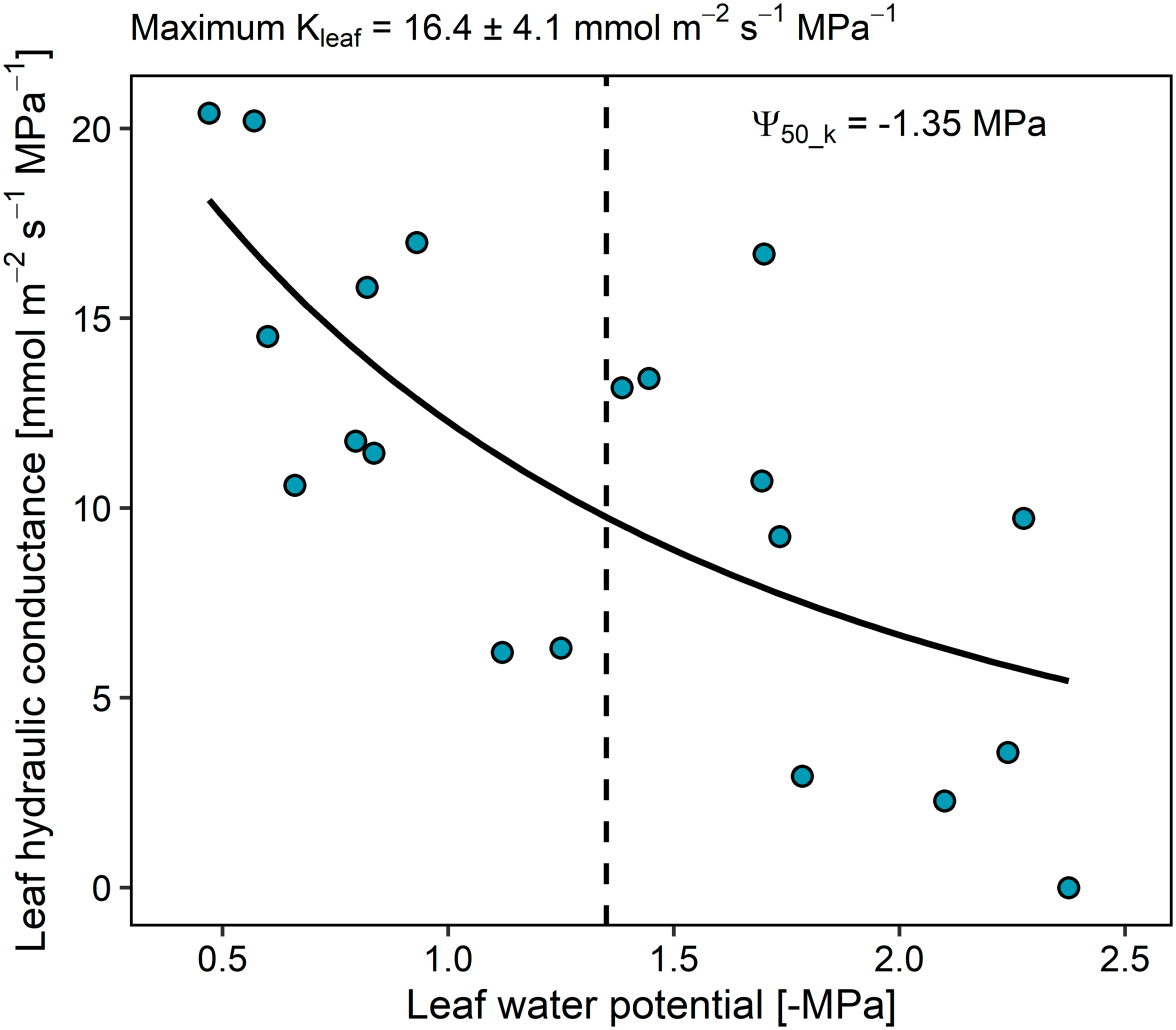
Changes in whole leaf hydraulic conductance as a function of leaf water potential as measured in plants deprived of water and subjected to progressive water stress. The solid line represents the interpolation of the experimental points, while the dashed vertical line indicates the leaf water potential value inducing 50% reduction of leaf hydraulic conductance compared to the maximum value (Ψ_50_k_).

The whole leaf hydraulic conductance (K_leaf_), as estimated based on the rehydration kinetic technique, averaged at 16.4 mmol s^−1^ m^−2^ MPa^−1^ in well hydrated plants (Ψ_leaf_ around −0.5 MPa). Upon dehydration, K_leaf_ declined progressively reaching values close to 0 at Ψ_leaf_ of about −2.5 MPa (Figure 2), while Ψ_50_k_ was −1.35 MPa (Figure 2).

**FIGURE 3:**
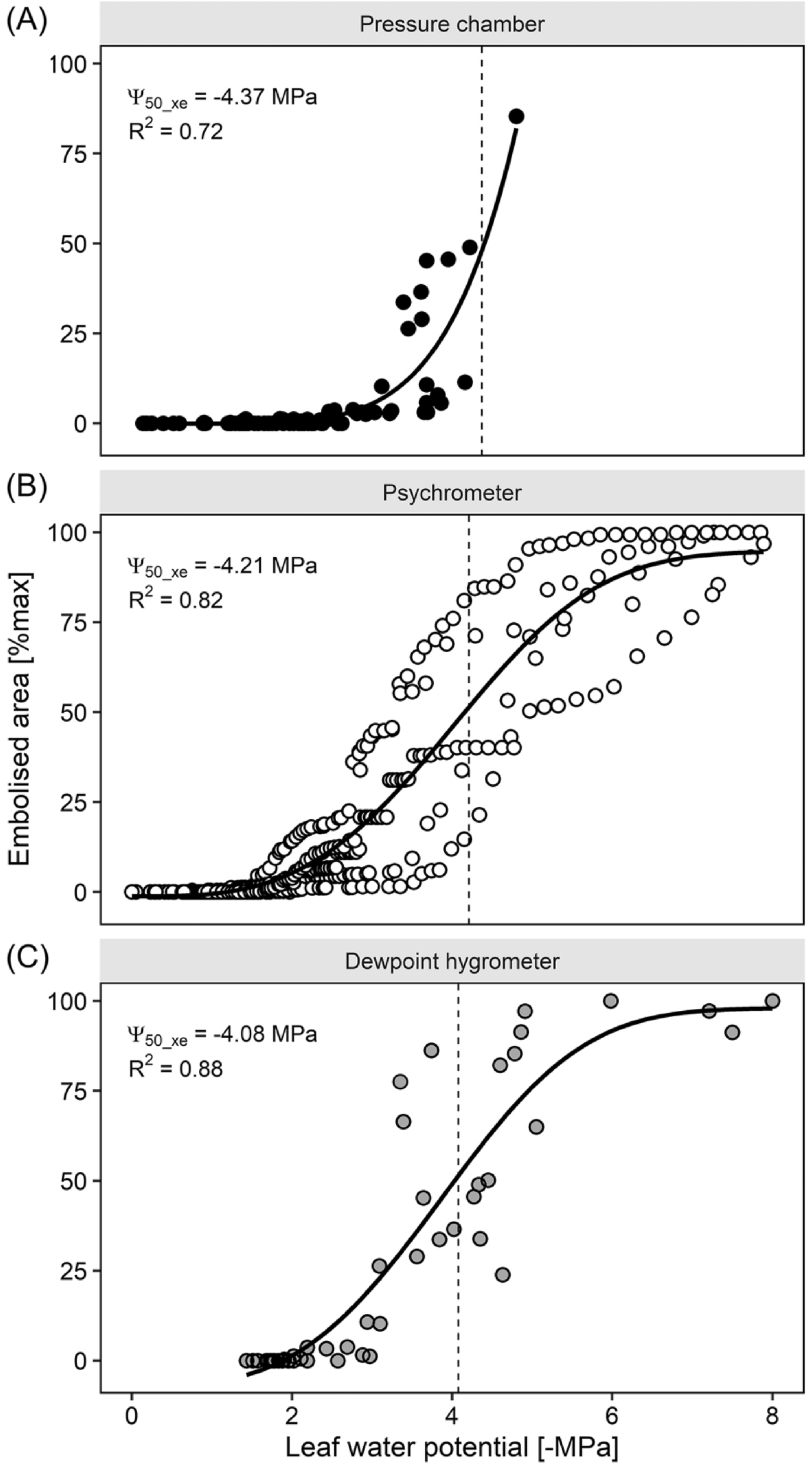
Vulnerability curves reporting the progressive increase of percentage embolised leaf area as measured with the optical method, as a function of leaf water potential during progressive plant dehydration. Leaf water potential was measured with three different methods i.e. (A) pressure chamber, (B) leaf psychrometer and (C) dewpoint hygrometer. Solid lines represent the interpolation of experimental points, while the dashed vertical lines indicate the leaf water potential values inducing 50% leaf xylem embolism (Ψ_50_xe_).

**FIGURE 4:**
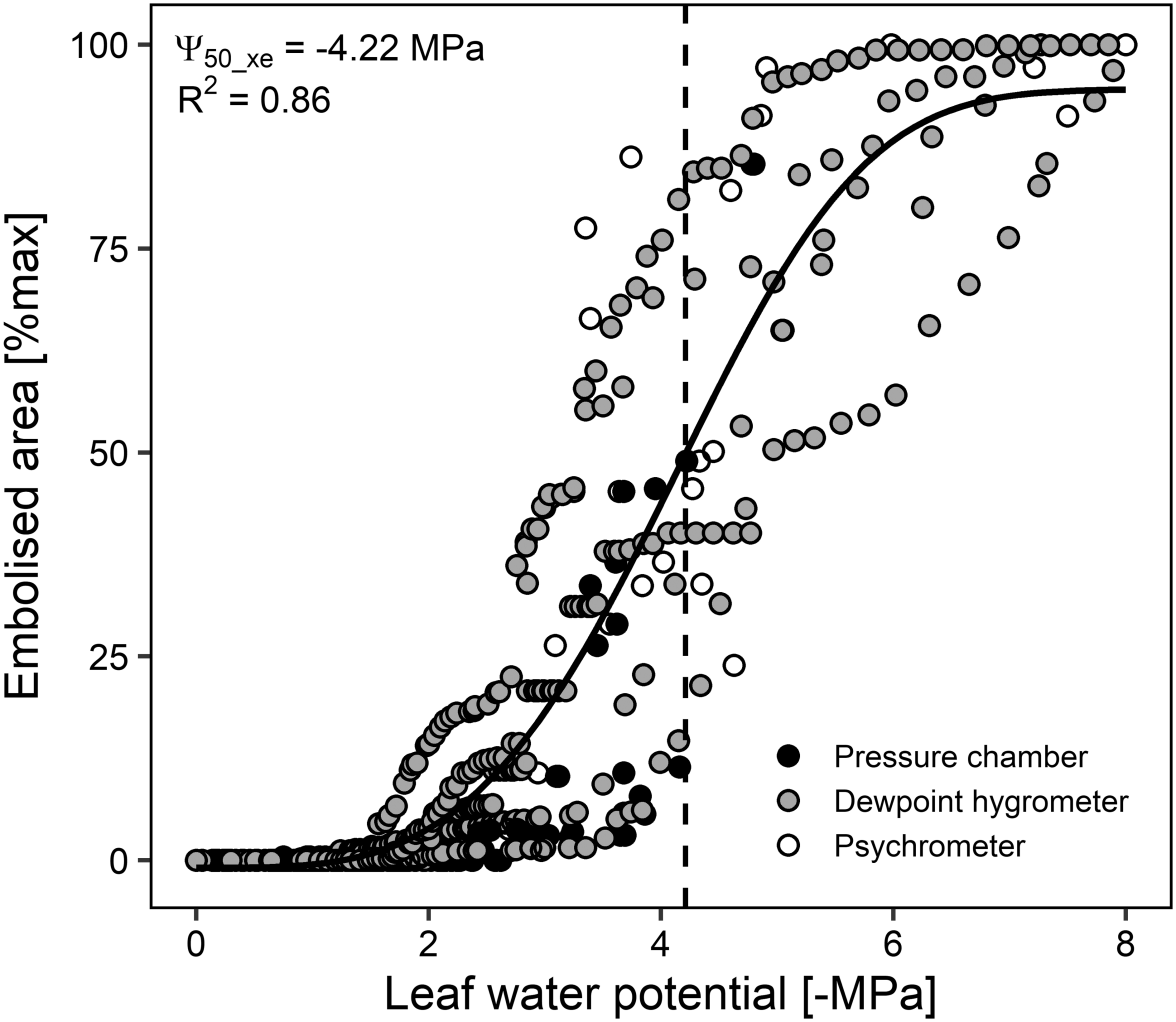
Vulnerability curve reporting the progressive increase of percentage embolised leaf area as measured with the optical method, as a function of leaf water potential during progressive plant dehydration. Leaf water potential was measured with three different methods i.e. pressure chamber, dewpoint hygrometer and leaf psychrometer (see Figure 3) and all data were pooled together. The solid line represents the interpolation of experimental points, while the dashed vertical line indicates the leaf water potential values inducing 50% leaf xylem embolism (Ψ_50_xe_).

**FIGURE 5:**
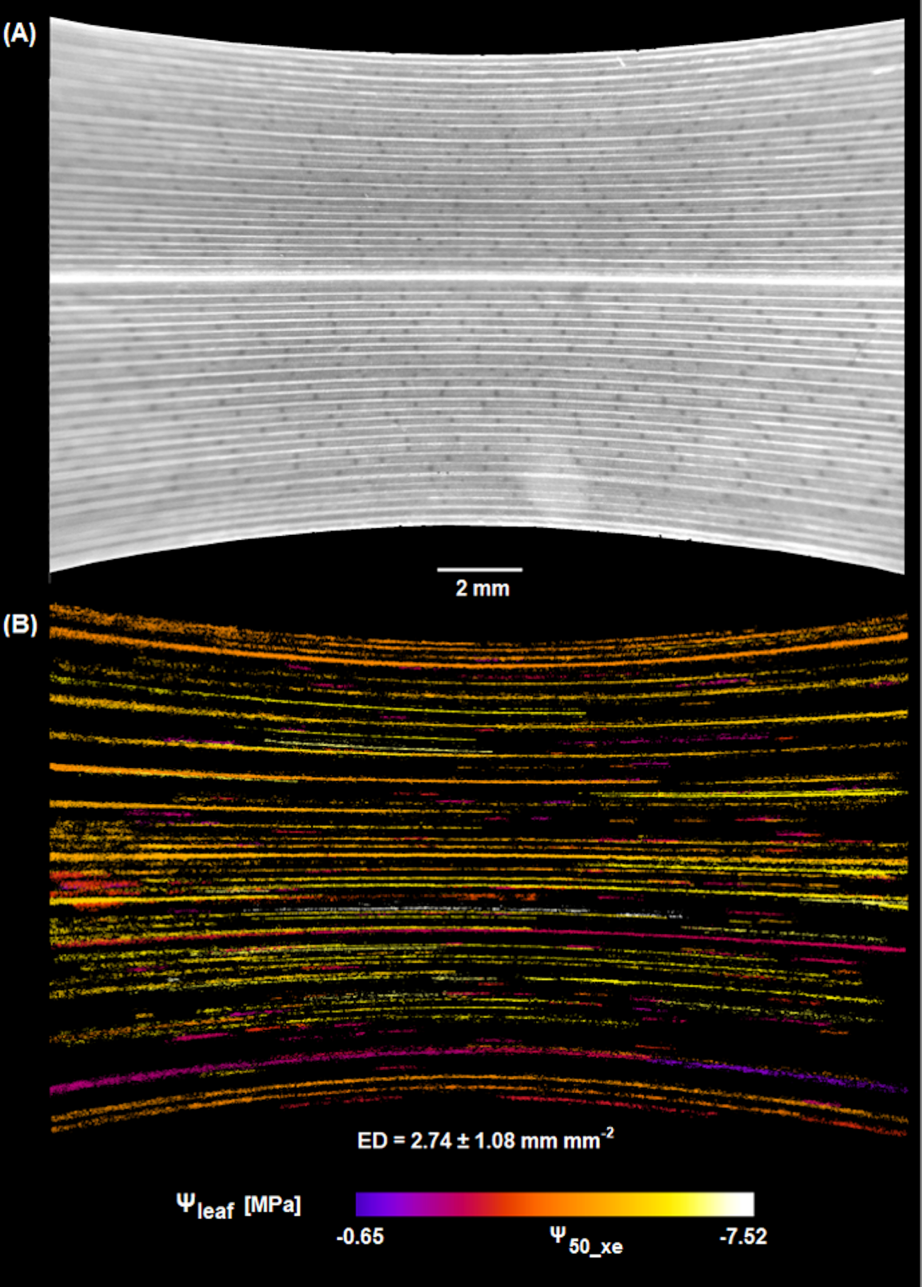
Images of leaves, as obtained with different techniques. (A) Example of an image taken using the optical technique. (B) Elaboration of images taken during progressive leaf dehydration; coloured pixels show cumulative embolisms over time, with a colour scale used to identify the water potential (Ψ_leaf_) at which events occurred (see colour scale on the right). Ψ_50_xe_ indicates the colour corresponding to 50% cumulated vein embolism. Total embolized vein density is also reported (ED, see also Figure S1).

We quantified the vulnerability to xylem embolism of the leaf veins using the optical method and a combination of techniques to estimate leaf water potential during progressive leaf dehydration, i.e. leaf psychrometer, dew‐point hygrometer or pressure chamber. The relationship between cumulative embolized area, expressed as a percentage of the maximum reached at the end of the dehydration, and Ψ_leaf_ was not different among the different methods (Figure 3, Table S1). Hence, all the data points were pooled together in a single plot (Figure 4). Optical measurements revealed that no or very few embolism events occurred at Ψ_leaf_ values above −2.0 MPa. Below this critical value, embolism started accumulating primarily in the major veins and some minor longitudinal veins (Figure 5), reaching maximum extension at Ψ_leaf_ between −6.0 and − 8.0 MPa when total ED reached a value of 2.7 ± 1.1 mm mm^−2^. Based on the sigmoidal model used to calculate the relationship between the percentage of embolized vein area and Ψ_leaf_, it was possible to calculate the water potential value inducing 50% xylem embolism in the vein xylem (Ψ_50_xe_), which averaged −4.22 MPa.

**FIGURE 6:**
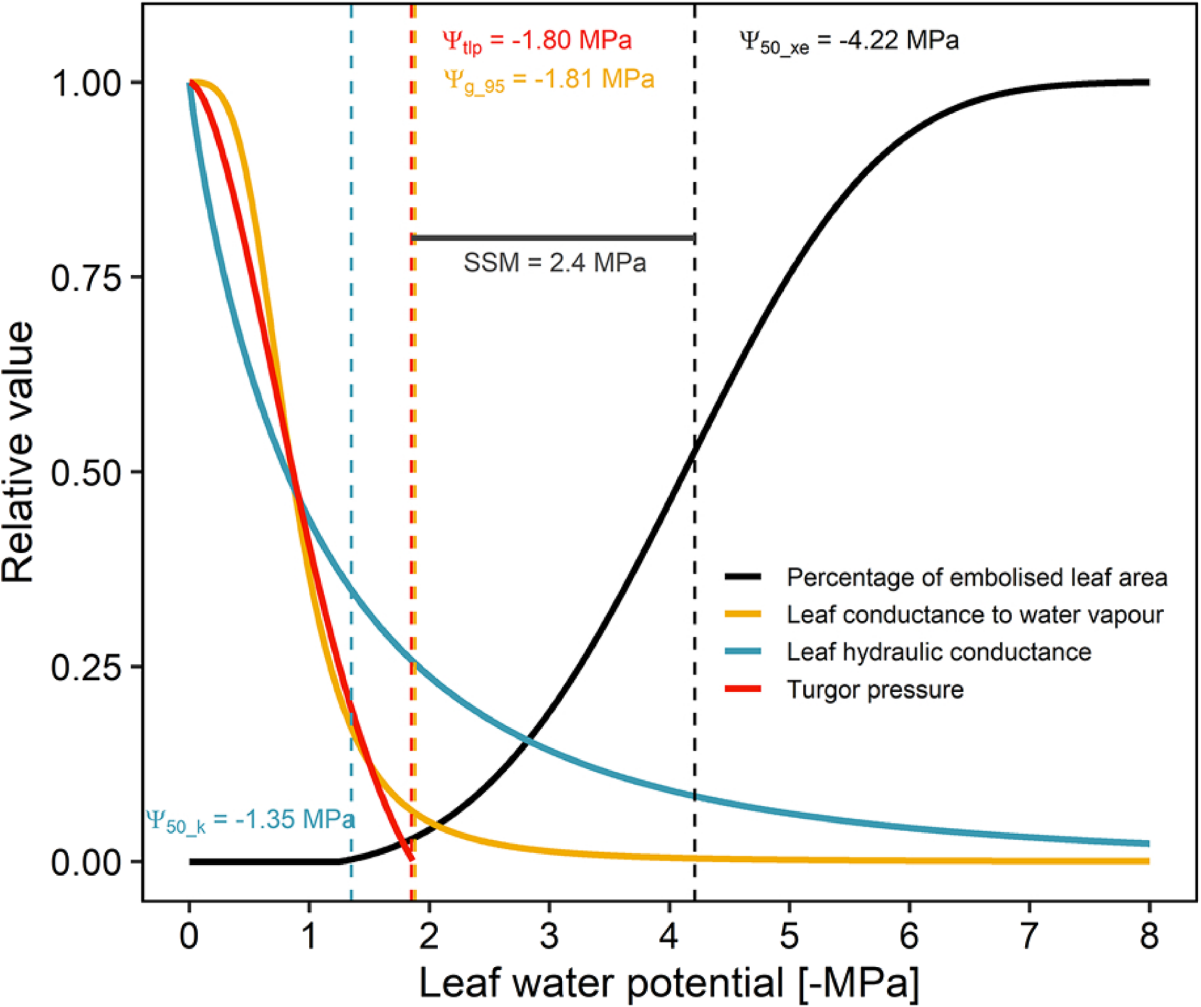
Relative changes of leaf vein embolism level (black line), leaf conductance to water vapour (yellow line), leaf hydraulic conductance (blue line) and turgor pressure (red line) as a function of leaf water potential during dehydration of plants of *Cyperus alternifolius*. Vertical dashed lines indicate the leaf water potential value inducing 50% xylem embolism (Ψ_50_xe_), 95% reduction of leaf conductance to water vapour (Ψ_g_95_), 50% reduction of leaf hydraulic conductance (Ψ_50_k_), or full turgor loss (Ψ_tlp_). The horizontal black line indicates the stomatal safety margin (SSM) as the difference between Ψ_g_95_ and Ψ_50_xe_.

Figure 6 reports the sequence of physiological events occurring during progressive decrease of leaf water potential, with a focus on stomatal responses and embolism build‐up, and related critical Ψ_leaf_ thresholds. Progressive stomatal closure started at Ψ_leaf_ < −1.2 MPa, reaching 50% of control values at Ψ_leaf_ = −1.4 MPa, i.e. a value corresponding to 50% loss of K_leaf_. Almost complete stomatal closure occurred at Ψ_leaf_ values corresponding to Ψ_tlp_ and to a 70% loss of K_leaf_, but before the onset of xylem embolism in the leaf vein system, that reached 50% only at Ψ_leaf_ < −4 MPa. This allowed to calculate the stomatal safety margin (SSM) which turned out to be 2.4 MPa.

## 4 DISCUSSION

Our comprehensive set of measurements of leaf hydraulics, water relations and gas exchange parameters in *C. alternifolius* offers a somehow unexpected view of the hydraulic safety and efficiency of this wetland plant and opens interesting perspectives towards the possibility of selecting crops combining both high productivity and high drought tolerance.

As expected on the basis of its ecological features, the study species displayed quite high gas exchange rates under well‐watered conditions. Leaf conductance to water vapour was about 400 mmol m^−2^ s^−1^ and this likely translated into high potential rates of CO_2_ fixation, due to the well‐known close relationship between maximal gas exchange rates and photosynthetic capacity (Haworth et al., 2021). The peak g_leaf_ value recorded in *C. alternifolius* is close to those reported for major and highly productive crops (Hoshika et al., 2018). This is not surprising when considering that the species often occupies high resource environments where rapid growth and biomass accumulation is a critical competitive strategy. Indeed, it is well know that wetland plants generally display a set of functional traits associated with fast resource acquisition (Pan et al., 2020). In line with high g_leaf_ values, we also found *C. alternifolius* to display high values of maximum leaf hydraulic efficiency, approaching 17 mmol s^−1^ m^−2^ MPa^−1^ i.e., close to the maxima recorded for species from tropical moist forests as well as for the most productive crops (Sack & Holbrook 2006; Nardini & Luglio, 2014). This likely explains the high gas exchange rates recorded, considering that maximum photosynthetic capacity and transpiration rates are strictly limited by the efficiency of liquid water transport within the leaf to avoid excessive dehydration and water potential drop (Brodribb et al., 2005).

Upon exposure to drought stress, *C. alternifolius* promptly underwent partial and progressive stomatal closure that finally led to almost full cessation of gas exchange at leaf water potential corresponding to the full loss of turgor, as already observed for several different species (Waite et al., 2024). The Ψ_tlp_ and π_0_ values measured for the study species are in the range globally recorded for plants growing in temperate habitats, but significantly lower (i.e. more negative) than those typically recorded in crops or in other wetland plants (Bartlett et al., 2012; Sueltenfuss et al., 2020). The adaptive role of Ψ_tlp_ to cope with water shortage is well recognized, and the low value of this variable in *C. alternifolius* suggests moderate drought tolerance, which is apparently at odds with the ecological niche preferentially occupied by this species. On the other hand, the bulk modulus of elasticity (ε) is close to maximum values recorded at a global scale (Bartlett et al., 2012). Evidences for the adaptive role of the bulk modulus of elasticity are scant and contrasting, and the current view is that high ε in leaves arises as a consequence of mechanical reinforcement of cell walls that might have or might not have a mechanistic connection to drought tolerance (Bartlett et al., 2012; Nardini, 2022). In particular, thick cell walls of parenchymatic cells might be required to sustain high turgor pressure under full hydration when π_0_ is very negative.

Before full stomatal closure, the progressive reduction of g_leaf_ with decreasing Ψ_leaf_ was paralleled by progressive reduction of cell turgor as well as of whole leaf hydraulic conductance (Figure 6). The decrease in cell turgor and/or the progressive reduction in cell volume have been suggested to be key signals triggering ABA production at leaf level, and in turn ABA accumulation would lead to progressive stomatal closure (McAdam & Brodribb, 2016; Sack et al., 2018). In this sense, high ε might allow a rapid turgor drop even for small reductions of cell volume, i.e., small water loss, thus prompting guard cells to close before excessive dehydration. Other studies have suggested that stomatal closure might be induced by progressive loss of hydraulic efficiency, due to modifications of either the vascular or extra‐vascular hydraulic pathways (Scoffoni et al., 2018; Albuquerque et al., 2020). In *C. alternifolius*, we did not observe any significant embolism at water potential above −2.0 MPa, suggesting that the initial drop of K_leaf_ was not due to xylem impairment but rather to a reduction of hydraulic efficiency in the extra‐vascular pathway, as already observed for other species in several previous studies (Savi et al., 2016; Trifilò et al., 2016; Scoffoni et al., 2017; Scoffoni et al., 2018; Ocheltree et al., 2020). It should also be noted that progressive turgor loss might drive the reduction of K_leaf_ via the inactivation of aquaporins or modifications of the cell‐to‐cell connectivity (Scoffoni et al., 2023).

Xylem embolism events in the veins were initially observed at Ψ_leaf_ lower than −2 MPa, i.e. immediately below Ψ_leaf_ values triggering turgor loss, full stomatal closure and a 75% reduction in K_leaf_. This means that xylem embolism in the leaves of *C. alternifolius* does not occur as long as the plant is transpiring, as previously observed for other species (Martin‐StPaul et al., 2017). This finding also suggests that stomatal closure is set to prevent the risk of excessive water potential drop initiating a process of hydraulic failure due to embolism propagation in the xylem network. During continued dehydration, embolism progressively accumulated in leaf veins and 100% loss of xylem function was reached at Ψ_leaf_ < −6 MPa. No clear embolism pattern was observed, although the first embolism events were consistently observed in a major vein, either the central or a lateral one. In no case, embolism events could be observed in the cross veins. On this basis, the calculated Ψ_50_xe_ of the study species was −4.2 MPa, i.e., 2.4 MPa more negative than the Ψ_leaf_, inducing full turgor loss and complete stomatal closure. Such a wide stomatal safety margin likely makes this species relatively safe against the risk of catastrophic hydraulic failure during occasional severe drought (Jacob et al., 2022), possibly favoring prompt recovery of water status and gas exchange following rewetting (Bi et al., 2023). It should be noted that the final embolized vein density was lower than the total vein density obtained after leaf clearance (Figure S1), and no further embolism events were detected at lower Ψ_leaf_. This suggests that not all veins and embolism events can be adequately captured by the OV technique, or that several veins are even more resistant to xylem embolism than those that could be clearly visualized (Petruzzellis et al., 2020).

The Ψ_50_xe_ value found for *C. alternifolius* (−4.2 MPa) is surprisingly low, especially when compared with those reported by Alemán‐Sancheschúlz et al. (2020) for three other wetland plants i.e. *Canna indica* (−0.13 MPa), *Cyperus papyrus* (−0.18 MPa), and *Phragmites communis* (−0.38 MPa), based on vulnerability curves generated with an air‐injection method on short stem segments. Air‐injection methods have been demonstrated to generate artifacts and produce biased estimates of vulnerability to embolism, especially when applied to short stem segments in long‐vessel species (Martin‐StPaul et al., 2013; Petruzzellis et al., 2023). The exponential shape of vulnerability curves reported by Alemán‐Sancheschúlz et al. (2020) would confirm the occurrence of experimental artefacts (Cochard et al., 2013) generating erroneously high Ψ_50_xe_ values. Indeed, such values would translate into immediate and massive embolism build‐up as soon as plants open stomata and initiate transpiration, leading to a Ψ_leaf_ drop, which is clearly inconsistent with the need to ensure high water flow rates to sustain gas exchange and photosynthesis. We suggest that previously published values of vulnerability to xylem embolism of wetland plants should be re‐evaluated based on experimental techniques limiting the risk of artifacts. In this sense, the OV method appears as a useful tool to investigate the hydraulic features of this interesting functional group.

Besides comparisons with other wetland plants, it is very interesting to note that the Ψ_50_xe_ of our study species falls in the lowest end of values recorded for a set of different herbaceous plants (Lens et al., 2016), making *C. alternifolius* one of the most drought tolerant herbs among the relatively few tested species in this regard, and yet it displays high hydraulic efficiency. The association of high hydraulic efficiency and safety observed in this species is somehow rare and unexpected (Yao et al., 2021). For example, several important herbaceous crops are known for their high hydraulic efficiency and photosynthetic productivity, but these are also generally quite vulnerable to xylem embolism. Skelton et al. (2017) reported a Ψ_50_xe_ of about −1.5 MPa for *Solanum lycopersicum*, while values of about −2.5 MPa were reported for *Triticum aestivum* (Corso et al., 2020). More recently, Li et al. (2024) provided estimates of Ψ_50_xe_ ranging between −0.9 and − 1.2 MPa for different cultivars of *Zea mays*. These trade‐offs between hydraulic efficiency and safety have been considered a major obstacle toward selecting crop cultivars better equipped to cope with intensifying drought but yet assuring similarly high yield. The finding that these two apparently contrasting functional traits coexist in an herbaceous wetland plant encourages further investigations of the anatomical and physiological determinants of hydraulic efficiency/safety. It also highlights the value of functional investigations of under‐investigated wild species, potentially holding the secrets leading to the generation of crops for a warmer and drier future.

Future studies should investigate the hydraulic safety/efficiency trade‐off in other wetland plants with contrasting ecological requirements, to check whether the case of *C. alternifolius* is unique or rather represents a combination of traits with an adaptive value in periodically inundated habitats. Additionally, the precise anatomical or molecular features underlying the combination of desirable traits observed in our study species should be disentangled to identify promising targets for breeding and selection of herbaceous crops sharing high hydraulic efficiency and yet appreciable levels of hydraulic safety.

## AUTHOR CONTRIBUTIONS

L.N.B., M.T. and A.N. conceived the study and planned the experiments. L.N.B., M.T. and F.P. performed the experimental measurements, analysed the data and prepared the graphs and figures. L.N.B. and A.N. wrote the manuscript, with contributions and revisions from M.T. and F.P.

## FUNDING INFORMATION

The study was funded by the Italian Ministry for University and Research, in the frame of the project PRIN2020 EvoPlant (Project title: The biochemical and diffusive optimisation of photosynthesis: evolutionary implications for the development of climate resilient productive plants). FP was supported by the funding Programma Operativo Nazionale (PON) Ricerca e Innovazione D.M. 1062/21 – Contratti di ricerca, from the Italian Ministry of University and Research (MUR).

## Supporting information


**Table S1:** Mean values and associated 2.5% and 97.5% confidence intervals limits of the water potential at which 95% of stomatal closure occurred (Ψ_g_95_), the water potential at which the leaves lose 50% of the maximum leaf hydraulic conductance (Ψ_50_k_), the water potential inducing 50% of embolized xylem area as calculated with the three methods to measure leaf water potential, i.e. pressure chamber (Ψ_50_xe_ pressure chamber), dewpoint hygrometer (Ψ_50_xe_ dewpoint hygrometer) and psychrometer (Ψ_50_xe_ psychrometer), as well as pooling all data together (Ψ_50_xe_ total).
**Figure S1**: Image of a cleared and stained leaf for determination of total leaf vein density.

## Data Availability

The data that support the findings of this study are available from the corresponding author upon reasonable request.
