# Supplementary material for: Better safe than sorry: the unexpected drought tolerance of a wetland plant (Cyperus alternifolius L.)
Source: Physiol Plant. 2024 Dec 26;177(1):e70027. doi: 10.1111/ppl.70027 (PMC11670444; doi:10.1111/ppl.70027)
Supplement: Supplementary file 1 — Table S1: Mean values and associated 2.5% and 97.5% confidence intervals limits of the water potential at which 95% of stomatal closure occurred (Ψg_95), the water potential at which the leaves lose 50% of the maximum leaf hydraulic conductance (Ψ50_k), the water potential inducing 50% of embolized xylem area as calculated with the three methods to measure leaf water potential, i.e. pressure chamber (Ψ50_xe pressure chamber), dewpoint hygrometer (Ψ50_xe dewpoint hygrometer) and psychrometer (Ψ50_xe psychrometer), as well as pooling all data together (Ψ50_xe total). Figure S1: Image of a cleared and stained leaf for determination of total leaf vein density. [file PPL-177-e70027-s001.docx]

Table S1: Mean values and associated 2.5% and 97.5% confidence intervals limits of the water potential at which 95% of stomatal closure occurred (Ψ_g_95_), the water potential at which the leaves lose 50% of the maximum leaf hydraulic conductance (Ψ_50_k_), the water potential inducing 50% of embolized xylem area as calculated with the three methods to measure leaf water potential, i.e. pressure chamber (Ψ_50_xe_ pressure chamber), dewpoint hygrometer (Ψ_50_xe_ dewpoint hygrometer) and psychrometer (Ψ_50_xe_ psychrometer), as well as pooling all data together (Ψ_50_xe_ total).

|  |  | 2.5% CI | 97.5% CI |
| --- | --- | --- | --- |
| Ψ_g_95_ | -1.81 | -1.64 | -2.10 |
| Ψ_50_k_ | -1.35 | -1.00 | -1.73 |
| Ψ_50_xe_ pressure chamber | -4.37 | -4.16 | -5.42 |
| Ψ_50_xe_ dewpoint hygrometer | -4.08 | -3.73 | -4.42 |
| Ψ_50_xe_ psychrometer | -4.21 | -4.03 | -4.38 |
| Ψ_50_xe_ total | -4.22 | -4.12 | -4.40 |

**Equation Figure 1**:

$y=c+ \frac{d-c}{1+ {exp(b\left( \log\left( x \right)-e \right))}^{f}}$

Parameters:

| Parameter | Estimate |
| --- | --- |
| b | -3.06 |
| c | -0.05 |
| d | 0.39 |
| e | 0.82 |
| f | 3.59 |

R^2^ model: 0.23

**Equation Figure 2:**

$y= \frac{100}{1+\exp(a\left( x-b \right))}$

Parameters:

| Parameter | Estimate |
| --- | --- |
| a | 1.46 |
| b | 1.97 |

R^2^ model: 0.50


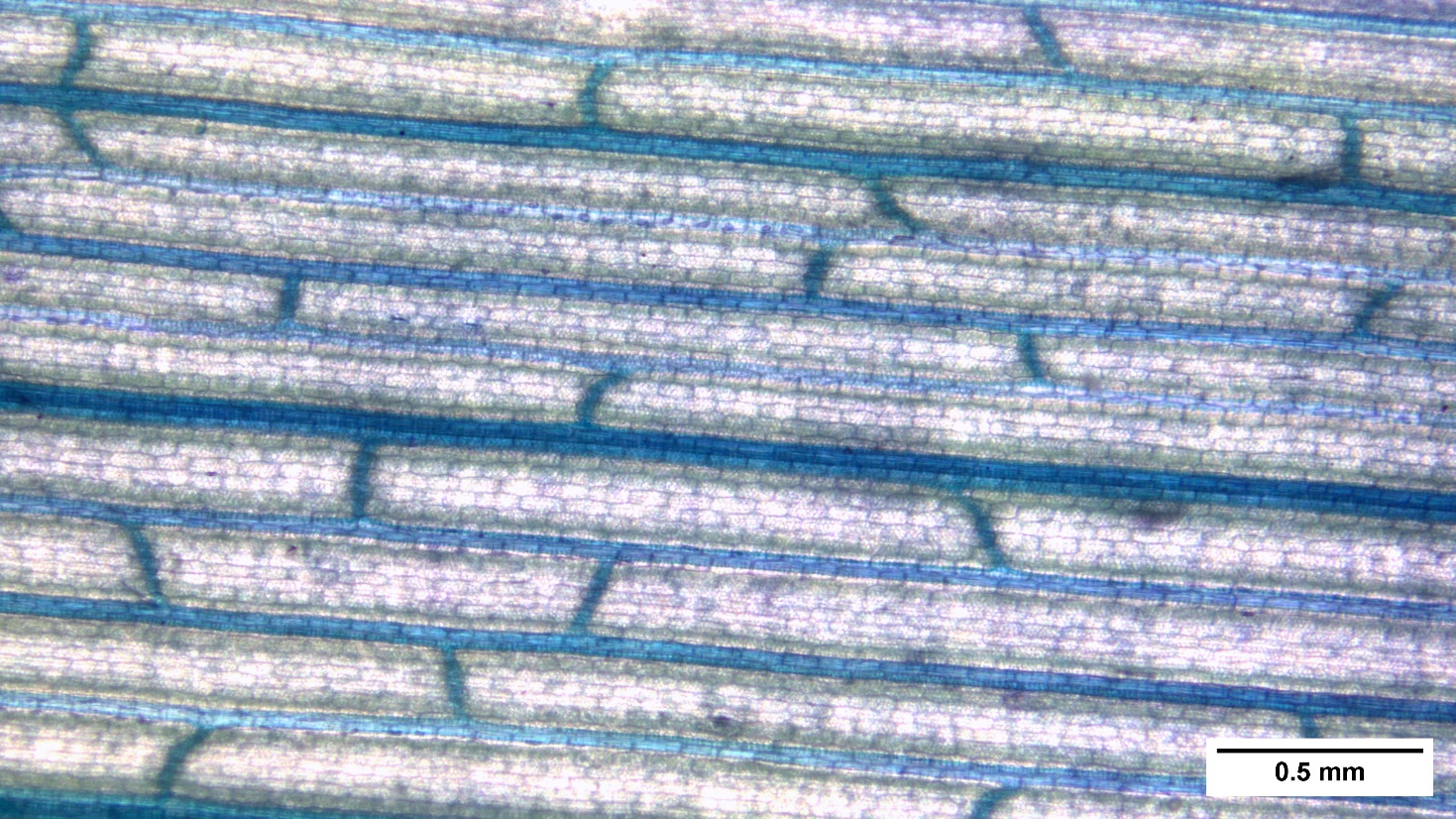


Fig. S1: Image of a cleared and stained leaf for determination of total leaf vein density.
